# Methodology of predicting novel key regulators in ovarian cancer network: a network theoretical approach

**DOI:** 10.1186/s12885-019-6309-6

**Published:** 2019-11-21

**Authors:** Md. Zubbair Malik, Keilash Chirom, Shahnawaz Ali, Romana Ishrat, Pallavi Somvanshi, R. K. Brojen Singh

**Affiliations:** 1 School of Computational & Integrative Sciences, Jawaharlal Nehru University, New Delhi, 110067 India; 2 Centre for Interdisciplinary Research in Basic Sciences, Jamia Millia Islamia, New Delhi, 110025 India; 3 Department of Biotechnology, TERI University, New Delhi, 110070 India

## Abstract

**Background:**

Identification of key regulator/s in ovarian cancer (OC) network is important for potential drug target and prevention from this cancer. This study proposes a method to identify the key regulators of this network and their importance.

**Methods:**

The protein-protein interaction (PPI) network of ovarian cancer (OC) is constructed from curated 6 hundred genes from standard six important ovarian cancer databases (some of the genes are experimentally verified). We proposed a method to identify key regulators (KRs) from the complex ovarian cancer network based on the tracing of backbone hubs, which participate at all levels of organization, characterized by Newmann-Grivan community finding method. Knockout experiment, constant Potts model and survival analysis are done to characterize the importance of the key regulators in regulating the network.

**Results:**

The PPI network of ovarian cancer is found to obey hierarchical scale free features organized by topology of heterogeneous modules coordinated by diverse leading hubs. The network and modular structures are devised by fractal rules with the absence of centrality-lethality rule, to enhance the efficiency of signal processing in the network and constituting loosely connected modules. Within the framework of network theory, we device a method to identify few key regulators (KRs) from a huge number of leading hubs, that are deeply rooted in the network, serve as backbones of it and key regulators from grassroots level to complete network structure. Using this method we could able to identify five key regulators, namely, AKT1, KRAS, EPCAM, CD44 and MCAM, out of which AKT1 plays central role in two ways, first it serves as main regulator of ovarian cancer network and second serves as key cross-talk agent of other key regulators, but exhibits disassortive property. The regulating capability of AKT1 is found to be highest and that of MCAM is lowest.

**Conclusions:**

The popularities of these key hubs change in an unpredictable way at different levels of organization and absence of these hubs cause massive amount of wiring energy/rewiring energy that propagate over all the network. The network compactness is found to increase as one goes from top level to bottom level of the network organization.

## Background

Ovarian cancer (OC) is an assorted cancer that begins in an ovary. Although most of OC’s are non-metastatic or having low potential to migrate, ovarian tumors can metastasize to other parts of the body and can be fatal. In 2016, it was reported 22,280 will receive a new diagnosis of ovarian cancer and 14,240 women will die from ovarian cancer [1, 2]. As the eighth-most common cause of death, OC is considered as the ‘silent killer’ due to lack of symptoms in its initial stages [1, 2]. In the preceding few decades, genetic studies have retrieved some genetic alterations that are also crucial in the pathogenesis of ovarian cancer. The swift growth of next-generation sequencing technologies recently has allowed the possibility for identifying many somatic alterations (genetic) in OC. These somatic alterations can be assessed as the passengers, which on the other hand pose challenge in classifying any cancer [3]. Identification of molecular drivers associated with a specific cancer type/sub-type is crucial and at the same time important for understanding its heterogeneity to seek treatment. In recent studies, [4] network based calculation have been implicated on multiple data sources to identify driver genes, including copy number variation, microRNA expression [4].

Epithelial ovarian cancers (EOC) remained the most lethal cancer among developed nations. From its different sub-types mEOC (mucinous epithelial ovarian cancers) represent approx. 3% of EOC. It can be divided into the more common, type II (aggressive) and type I (slow growing) cancers [5]. Low-grade i.e. type I is present in young women and have a high prevalence of KRAS and BRAF mutations, but low in relation to Tp53 mutations (that characterize to type II). These prevalent mutations qualify as a favourable prognostic factor for type I EOC. While at the same time identifying MAPK, mutations are useful in guiding clinical treatment [6]. Most of the new cases present with advanced stage of disease have initial treatment which consists of a cyto-reductive surgery and chemotherapy [7]. While the patients that develop advanced EOC are the ones having pre-clinical diminution after primary therapy. Though, long term cure resides in the patients exposed to multiple chemotherapeutic agents [8]. Thus, the identification of malignant ascites is the most common consequence of EOC. It causes significant symptoms and impact on the patient’s life, more than ever in cases where women have regular ovarian cancer [9].

 The current paradigm for finding out OC revolves around the identification of critical regulators in the Transcriptional factor (TF) networks present in OC cells. As these TFs might be important therapeutic targets [10]. To understand the mechanism and predict the complex interaction within the complex biological network and how numerous basic functions are performed by the organization of the components between them. The large scale data from the omics have interestingly been used to map genes with specific diseases [11]. Network theory has been presented to bring a significant approach to understanding the complex systems dynamics and topo- logical properties, to co-relate to their functional modules [12]. The large number of the existing networks fall into different type of network such as, hierarchical net- work, scale-free, random and small world [13]. Amongst these type of network, the hierarchical type network finds special attention from biologists due to its sparsely distributed hubs that regulates the ovarian cancer network as well as also appearance of modules [13, 14]. The appearance of modules in this type of network is of significance to us because they can correlate to independent functional factor in the network which comply with their own laws [13]. Therefore, we tend to focus our study on network of ovarian cancer, which is developed from experimentally verified known genes of ovarian cancer and their interactions to analyze potential key regulatory (KRs) genes which may serve as potential target genes. Therefore, We also aimed to explain its topological properties from which we tend to attempt to conclude potential key regulators among that a few are of elementary effect, their regulating as well as activities mechanism [14].

## Methods

### Workflow of construction of ovarian cancer network and techniques of analysis

**Fig. 1:**
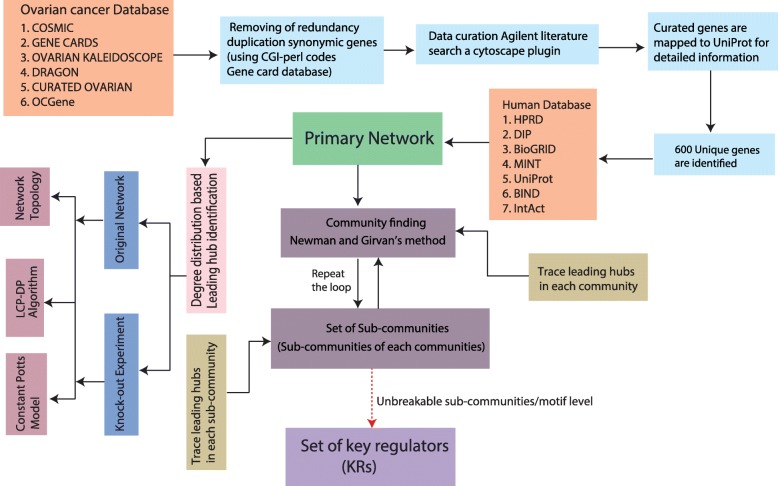
Schematic diagram of the workflow of the methods implemented in the study of ovarian cancer network

The detailed workflow of the ovarian cancer network and analysis is given in Fig. 1 and detail techniques are given in the Additional file 1. We describe briefly the workflow of the analysis below.

### Acquisition of ovarian cancer data

We extracted 6 (sets) list of ovarian cancer genes from 6 highly cited cancer database and then integrated the common ovarian cancer gene from all the list in order to retrieve a list of only experimentally verified gene of ovarian cancer. The different resources used are COSMIC database, Gene cards database, Ovarian kaleidoscope database, Dragon database of ovarian cancer, curated ovarian database and OC- Gene database, which are focused on different aspects of cancer biology (see Additional file 1). We have assimilated 2000 genes from the mentioned repositories. This list of genes is subjected to CGI code written in Perl to remove duplication genes in terms of redundancy of names as well as aliases used for gene names (for details see Additional file 1). This method allows to filter unique 660 genes out of 2000. The list of the genes is further curated manually as well as using Cytoscape 3.7.1 plugin, then mapped to UniProt (January 2016) and finally we arrive at 600 genes (for details see Additional file 1).

### Network construction of the curated genes

We followed the idea of one protein one gene concept to construct primary protein- protein interaction network using the curated list of 600 genes using GeneMANIA App [15] and verified and uploaded the file in Cytoscape [16] using Cytoscape 3.7.1. The constructed network now consists of 4818 nodes having 16320 possible connections among them (for details see Additional file 1).

### Method to identify key regulators

We first find out a list of the first seventy (one can vary the number) leading hubs characterized by the degree distribution of the complete network. Now communities of the network are extracted using Newman and Grivan’s community finding method [17] which is the level of organization of communities of network (say first level of network organization). We then trace the leading hubs in each community and isolated hubs are discarded. Now sub-communities of all the communities are found out using the same community finding method mentioned above and the leading hubs are traced in each sub-community at this second level of organization followed by rejection of isolated or truncated hubs. The process is repeated until a situation is reached where the smaller communities can no longer breakable (most probably at motif level) by the method and trace the hub genes reach at this level. The set of the leading hubs reach at this level (lowest level) is termed as a set of *key regulators*. These KRs can be considered as the backbone of the network, which are deeply rooted and involved at each level of the organization from top level to bottom level and vice versa.

### Topological analyses of the networks

The topological properties of ovarian cancer PPI network are characterized by degree distribution, clustering co-efficient, connectivity and centrality (betweenness, closeness and eigen-vector) measurements (for details see Additional file 1). We use these parameters to understand topological changes when the network is perturbed [14, 18, 19].

### Tracking of genes in ovarian cancer network

The most influential genes in the OC network were identified first through calculating the centrality measures. Since, higher degree nodes have higher centrality values, top 70 highest degree nodes were considered among the hub nodes of the network for tracing the key regulators which may play important role in regulating the network. Then tracing of nodes from the primary network up to motif level was done on the basis of representation of the respective nodes (proteins) across the sub modules obtained from Louvain method of community detection/ clustering. Finally, the hub-nodes (proteins) which were represented at the modules at every hierarchical level were considered as key regulators of the OC network.

### Knock-out experiment

The role KRs can be investigated by performing knock-out experiment of the net- work and studying the variations in topological properties of OC cancer PPI net- work. In the this experiment the KRs are systematically removed from ovarian cancer network and calculate topological properties of ovarian cancer and sub- communities at each level of organization of network to compare with the original ones before KRs are removed (for details see Additional file 1) [14].

### Network compactness estimation

The degree of how strongly the nodes are linked in a network and its associated com- munities and the sizes can be estimated by LCP-DP (local community paradigm- decomposition plot) algorithm [20] (for details see Additional file 1). We used this method to analyze organizational behavior of the ovarian cancer network.

### Constant Potts model

The energy distribution in a network can be estimated by using constant potts model [21] (for details see Additional file 1). This technique was applied to understand the importance of KRs in a network and their regulating activities.

### Survival analysis

Survival analysis of the key regulators were performed using kemplotter [22, 23]. All the datasets were taken along with TCGA dataset for the analysis with total sample size of overall survival (*n* = 1657) using ovarian cancer probset. Overall survival probabilities were plotted on Y axis and time of survival in months were plotted on *X* axis and logrank *p* value <0.05 was taken as statistically significant value between the low and high expressions of genes.

### Gene ontology and Pathways analysis

We perform GO and pathways analysis by using DAVID web server (Database for Annotation, Visualization and Integrated Discovery) [24, 25].

## Results

### Ovarian cancer network follows hierarchical scale free features

**Fig. 2:**
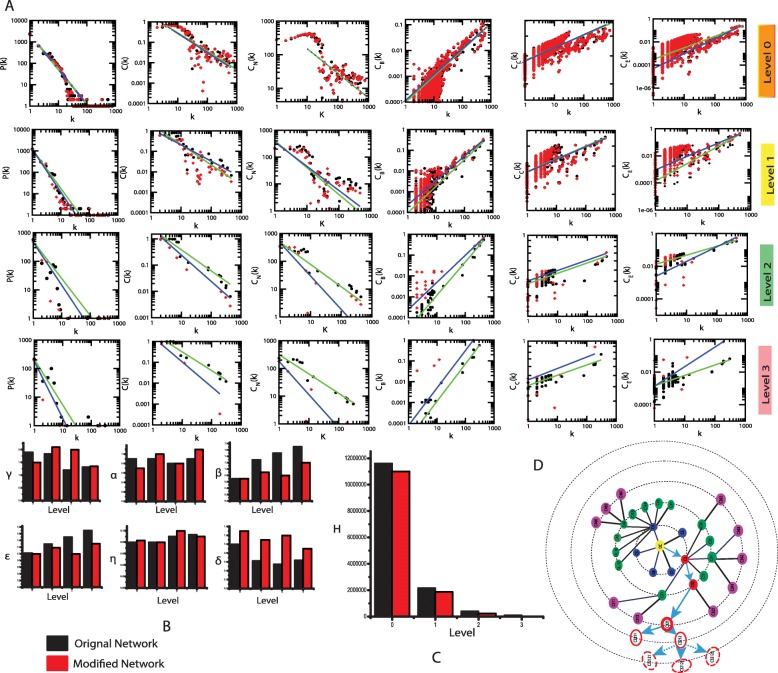
Topological properties of the ovarian cancer network. **a**. The behaviours of degree distributions (*P*(*k*), clustering co-efficient (*C*(*k*)), neighbourhood connectivity (*C*_*N*_(*k*)), betweenness (*C*_*B*_(*k*)), closeness (*C*_*C*_(*k*)) and eigen-vector (*C*_*E*_(*k*)) measurements as a function of degree k for original and five key regulators knock-out network at different levels of organization. **b**. The changes in the exponents of the six topological parameters due to key regulators knock-out experiment. **c**. Energy distribution in the network quantified by Hamiltonian calculation as a function of network levels. **d**. Changes in the network modules/sub-modules due to five key regulators knock-out experiment. The dotted modules/sub-modules are the break-down modules/sub-modules

The ovarian cancer network, which is the proposed complex regulatory network to be studied, is constructed from the experimentally verified seventy genes (see Methods and Table 1). The topological properties of this network, namely, probability of degree distribution *P* (*k*), neighborhood connectivity *C*_*N*_(*k*) and clustering co-efficient *C*(*k*) follows power law characters as a function of *k*. (Fig. 2 1^st^ row denote *Level* 0). The power law fits on the complex data sets of the topological variables of the ovarian cancer network are performed and conformed following a standard statistical fitting procedure suggested by Clauset *et al.* [26], where, all *p*-values (statistical) for all data sets, estimated versus 2500 random samplings, are establish to be ≥ 0.1 (critical value) and goodness of fits is establish to be ≤ 0.33 (Fig. 2 first row blue fitting line). The exponential values are retrieved from the power law fittings. For the entire ovarian cancer network, the results are summarized as follows,

1 $$ \Gamma \left(\mathrm{k}\right)\left[\ \begin{array}{c}{\Gamma}_1\\ {}{\Gamma}_2\\ {}{\Gamma}_3\end{array}\right]=\left[\begin{array}{c}P\\ {}C\\ {}{C}_N\end{array}\right]=\left[\begin{array}{c}{k}^{-\upgamma}\\ {}{k}^{-\upalpha}\\ {}{k}^{-\upbeta}\end{array}\right];\left[\begin{array}{c}\gamma \\ {}\alpha \\ {}\beta \end{array}\right]\longrightarrow \left[\begin{array}{c}2.16\\ {}0.9\\ {}0.67\end{array}\right] $$

**Table 1:** Gene Ontology Pathway Enrichment Analysis of level 5 communities of ovarian cancer network

S. No.	Community	Pathway	Identifier	Reference Pathway	Genes	*p*-value	Benjamini corrected p-value
1.	C32121	Rap1 signalling pathway	hsa04015	KEGG	PIK3CG, RASSF5, PLCB3, KRAS, RAC2, RASGRP2, RAC1, RALGDS	3.9E-5	5.5E-3
2.	C32122	PI3K-Akt signalling pathway	hsa04151	KEGG	IL2RB, IL2RA, PDGFB, PIK3CB, PPP2R5C, TCL1A, NR4A1, TCL1B, BAD, BCL2L11, AKT1, EIF4EBP1, TSC1, PDGFRB, RELN, MTCP1, NOS3, IL2RG, JAK3, IKBKB, THEM4, IL2	4.9E-11	7.3E-9
3.	C32123	Proteoglycans in cancer	hsa05205	KEGG	CDC42, ANK1, EZR, CD44, CAMK2G, RRAS, ITGB5, ITGA2, MSN, FLNC, CD63	2.3E-7	2.6E-5

These topological properties of the ovarian cancer PPI interaction network are very close to ideal hierarchical properties of the network whose values of the exponents are, *γ* ≈ 2.26 (mean-field theoretical value) [13], *α* = 1 [27, 28] and *β* = 0.5 [29]. This topological function Γ_*i*_; *i* = 1, 2, 3 satisfy the Mandelbrot’s classical definition of fractal [30], which is defined by the following self-affine process of any scale factor *λ*,

2 $$ \frac{\Gamma_i\left(\lambda k\right)}{\Gamma_i(k)}={\lambda}^D;\mathrm{D}=\left[\ \begin{array}{c}{\mathrm{D}}_1\\ {}{\mathrm{D}}_2\\ {}{\mathrm{D}}_3\end{array}\right]=\left[\begin{array}{c}-\gamma \\ {}-\alpha \\ {}-\beta \end{array}\right] $$

where, *D*_*i*_ corresponds to fractal dimension of *i*^*th*^ topological parameter. Hence the ovarian cancer network follows the fractal features or the hierarchical scale free. The negative values in fractal dimension indicate the enrich randomness in the network organization with sample variability [31]. Since the *c*_*N*_(*k*) has a negative power in k i.e. *β* = 0.67, the ovarian cancer network exhibits disassortivity nature which means that the *rich-club* formation of a large number of the leading/major hubs in the network is unlikely [29].

The centrality measurement, such as, closeness *C*_*C*_, betweenness *C*_*B*_ and eigen-value centrality *C*_*E*_, characterize the importance of the hubs, their regulating mechanisms (see Methods) and obey the following power law behaviors (Fig. 2 first row),

3 $$ \Lambda (k)=\left[\ \begin{array}{c}{\Lambda}_1\\ {}{\Lambda}_2\\ {}{\Lambda}_3\end{array}\right]=\left[\ \begin{array}{c}{C}_B\\ {}{C}_C\\ {}{C}_E\end{array}\right]=\left[\ \begin{array}{c}{k}^{\upvarepsilon}\\ {}{k}^{\upeta}\\ {}{k}^{\updelta}\end{array}\right];\left[\begin{array}{c}\varepsilon \\ {}\eta \\ {}\delta \end{array}\right]\longrightarrow \left[\begin{array}{c}1.15\\ {}0.083\\ {}1.11\end{array}\right] $$

The power law behaviour of the these *C*_*C*_, *C*_*B*_ and *C*_*E*_ (centrality measurements) are again confirmed and verified applying the Clauset *et al.* procedure of power law fitting, where, it is found that *p*-values to be greater than 0.1 and goodness of fit also greater than 3.5. Since only less numbers of higher degree nodes have great *C*_*C*_, *C*_*B*_ and *C*_*E*_ values, the number of greater regulating hubs, that can regulate the ovarian cancer network, is less. Hence, moderately low degree nodes (proteins/genes) dominate the network and therefore, the organization, functioning and regulation of the network are done mostly by these low degree proteins/genes. However, the sparsely distributed major/leading few hubs, that can be significant roles in maintaining as well as regulating the ovarian cancer network stability. Further, power law behavior of centrality measurements given by equation (3) follows the following self-affine process for any scale factor *c*,

4 $$ \frac{\Lambda_i(Ck)}{\Lambda_i(k)}={C}^{\mathbbm{D}};\mathbbm{D}=\left[\begin{array}{c}{\mathbbm{D}}_1\\ {}{\mathbbm{D}}_2\\ {}{\mathbbm{D}}_3\end{array}\right]=\left[\begin{array}{c}\epsilon \\ {}\eta \\ {}\delta \end{array}\right] $$

The positive values in $\backslash \mathrm{mathbbm}{\mathbbm{D}}_i$ indicate the distribution of the measurements in the network [31]. Since the topological variables show fractal nature, as evident from equations (2) and (4) respectively, the ovarian cancer network exhibits fractal or hierarchical scale free features.

### Key regulators in ovarian cancer network and properties

**Fig. 3:**
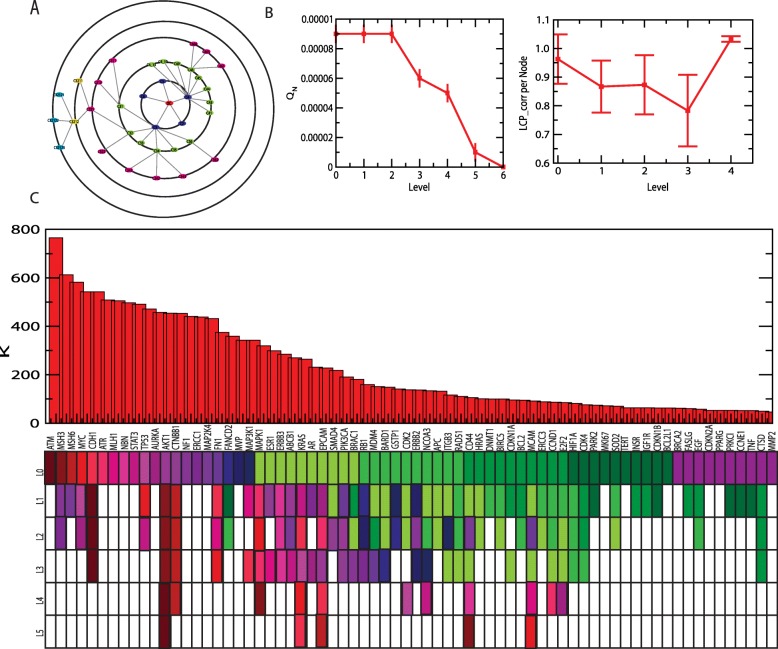
Identification of key regulators of ovarian cancer network. **a**. Organization of the modules/sub-modules of the network. **b**. Plots of *Q*_*N*_ and *LCP* − *corr* as a function of network level. **c**. Characterization of seventy leading hubs of the network by degree (*k*) distribution and identification of key regulators. Colour codes are popularities of the leading hubs

Since ovarian cancer PPI interaction network follows hierarchical scale free nature, the emergence of modules in the network is significant and therefore both these sparsely distributed few leading hubs regulate and modules, which is organize the network. Application of Newmann and Girvan’s standard community finding algorithm, the modular structure and their organization at different levels of organization were established (see Methods) [14, 17, 32]. Applying the present algorithm, the ovarian cancer network is established to be hierarchically organized through five various levels of organization shown in Fig. 3a). The corresponding to *Q*_*N*_ (modularity) and LCP, LCP-correlation per node as a function of levels of organization are established to decrease in concert goes from higher level to down level (Fig. 3b).

Depending on the degree of the nodes in the ovarian cancer network, the first seventy leading hubs are identified (Fig. 3c). The most appropriate question is whether these hubs are actual target genes that regulate network at fundamental level. Hence, we propose to define *key regulators* in the act of those proteins/genes that are deeply rooted from top level to bottom level of the ovarian cancer net- work organization and vice versa and play the role of backbone of ovarian cancer network organization (Fig. 3c). These KRs may or may not necessarily be major leading hubs in the ovarian cancer network, however randomly variation their popularities at different levels of organization. Removal of the major hubs doesn’t cause network disruption since the ovarian cancer network exhibits hierarchical characteristics. Though, the removal of key regulators from the ovarian cancer network can cause maximum perturbations both locally and globally in the network, specially at a deeper level of organization. Then the perturbations will propagate through different levels of organization’s top level to bottom of bottom level to top causing topological variation in the ovarian cancer network. Hence, we propose that these key regulators could be driver target genes of ovarian cancer.

**Fig. 4:**
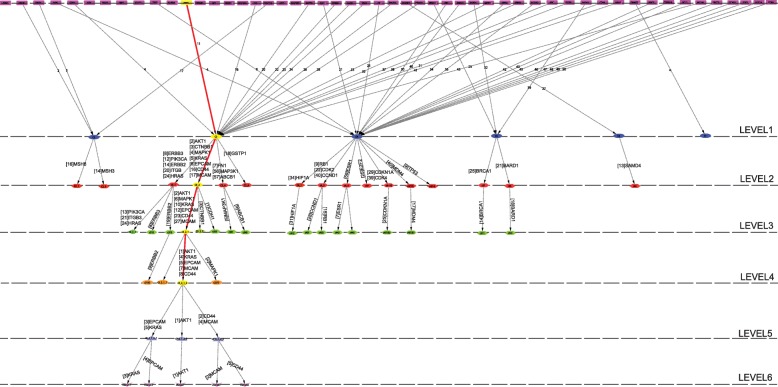
Tracing of key regulators of the network through different levels of the network

**Fig. 5:**
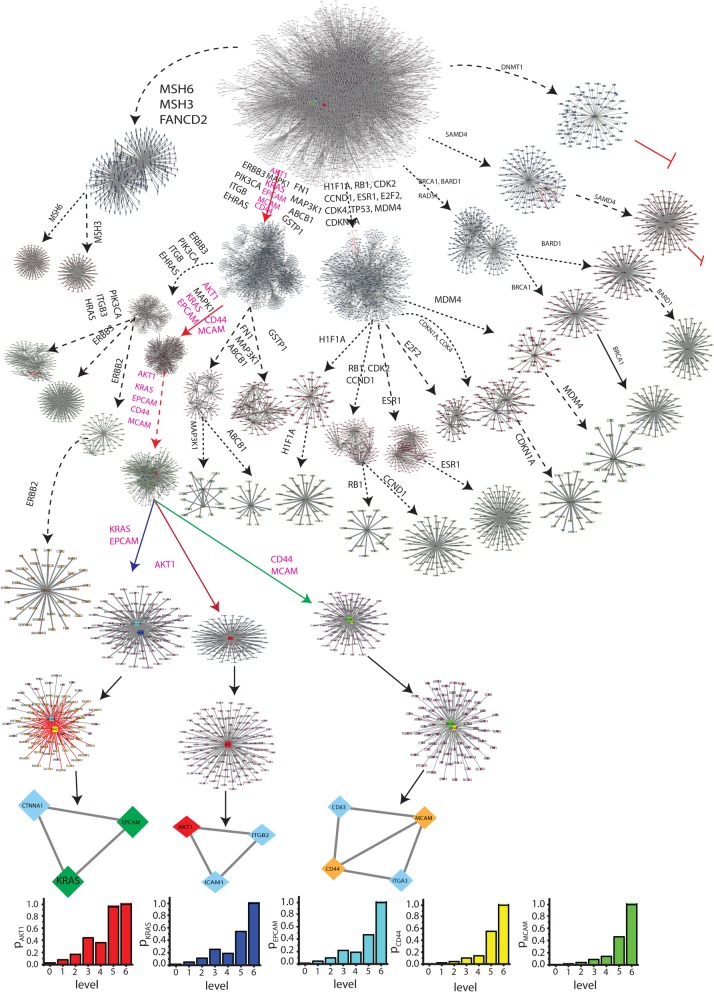
Network/modules/sub-modules at different network levels which accommodate leading hubs and key regulator regulators. The probability distributions of the key regulators as a function of level

Following the definition of key regulators, we could able to identify five KRs, namely, AKT1, KRAS, EPCAM, CD44 and MCAM (Fig. 3c, Figs. 4 and 5), that are key regulators (organizers) of the ovarian cancer network. Surprisingly, the top 11^*th*^ leading hubs aren’t obtained to be key regulators as they fail to reach till the deepest/lowest level of organization. Out of these five KRs few are at low profile/popularity (CD44 and MCAM) but could able to regulate till the bottom level of arrangement organization. Further, these key regulators start separating from one another after the third level; KRAS and EPCAM go together, AKT1 moves alone and CD44 and MCAM go together till motif (triangular type) level (Figs. 4 and 5). These key regulators perform as signal propagators from top level to bottom level and vice versa to control the inherent properties and network stability.

To understand regulating capability of total the five KRs, we define a probability *P*_*y*_(*x*^[*s*]^) of a KR *y* to have a number of links/edges *x*^[*s*]^ at level *s* out of the total number of links/edges *E*^[*s*]^ of the ovarian cancer network/module/sub-module in which that KR is accommodated, which is given by,

5 $$ {P}_y\left({x}^{\left[s\right]}\right)=\frac{x^{\left[s\right]}}{E^{\left[s\right]}},\forall {E}^{\left[s\right]}\ne 0 $$

The calculated *P*_*y*_ of all the five KRs exhibit increase in *P*_*y*_ as one goes from top level to bottom level (when s increases) and found that *P*_*y*_ → 1 as *s* → 5 (Fig. 5 lowest panels). This reveals that the regulating capability of each key regulator becomes more prominent at deeper levels of organization. Further, the inherent regulating capability of each key regulator ${P}_y^{\left[I\right]}$ can be approximately measured by calculating average over *P*_*y*_ as in the following,

6 $$ {P}_y^{\left[I\right]}=\frac{1}{M+1}\sum \limits_{s=0}^{M=5}{P}_y\left({x}^{\left[s\right]}\right) $$

The calculated values of ${P}_y^{\left[I\right]}$ shows that ${P}_{AKT1}^{\left[I\right]}>{P}_{KRAS}^{\left[I\right]}>{P}_{EPCAM}^{\left[I\right]}>{P}_{CD44}^{\left[I\right]}>{P}_{MCAM}^{\left[I\right]}$ . The inherent regulating capability of AKT1 is highest and that of ${P}_{MCAM}^{\left[I\right]}$ is lowest.

### Local perturbations driven by ovarian cancer key regulators

The knock-out experiment of five key regulators from ovarian cancer network could able to highlight the local perturbations driven by these KRs and their effect on global network properties. The removal of these key regulators from the complete ovarian cancer network brings significant variations in the topological properties of the ovarian cancer network (Fig. 2a first row), where, *γ* and *α* change significantly in complete network level (Fig. 2b), whereas *β* change slightly. Similarly, the variations in the measurements of the exponents of centrality (*ϵ*, *η* and *δ*) also show significant (Fig. 2b). Since, all the five KRs are present in a single module/sub- module up to third level of organization, we only consider that module/sub-module for the five KRs knock-out experiments (Fig. 2 second, third and fourth rows). It is noticeable from the variations in the exponents of topological parameters (Fig. 2 B) such as one goes to deeper level i.e. The ovarian cancer network perturbation increases as goes from top direction to down direction. After the third level, the removal of these KRs almost breakdown the sub-modules present in the remaining levels (deeper) (Fig. 2d). This demonstrate that local perturbation caused by five KRs together is maximum at deeper levels and propagates the perturbation through other levels from bottom to top.

In order to understand variation in energy distributions in corresponding ovarian cancer network and modules/sub-modules at different level of organization. Now we calculated Hamiltonian of respective ovarian cancer network and modules/sub-modules in five know-out experiment (Fig. 2c) (see Methods). If $\Delta  {H}_s={H}_s^{\left[O\right]}-{H}_s^{\left[R\right]}$ is the change in Hamiltonian functions due to removal of five KRs at level s, where *H*^[*O*]^ and *H*^[*R*]^ are the Hamiltonian functions for original and removed networks respectively and corresponding modules/sub modules, then we obtain,

Where, ${H}_s={H}_s^{\left[O\right]}$. This demonstrates that removal of key regulators causes excessive destructive of wiring energy/rewiring energy that is propagated over all the levels of ovarian cancer network organization.

7 $$ \Delta  {H}_s>0,{\forall}_s;\left\{\begin{array}{c}{\left.\frac{\Delta  {H}_s}{H_s}\longrightarrow 0\right\rceil}_{s:3\longrightarrow 0}\ \left( for\ s\le 3\right)\\ {}{\left.\frac{\Delta  {H}_s}{H_s}\longrightarrow 1\right\rceil}_{\forall s}\ \left( for\ s>3\right)\end{array}\right. $$

### Network compactness preserves self-organization in ovarian cancer

**Fig. 6:**
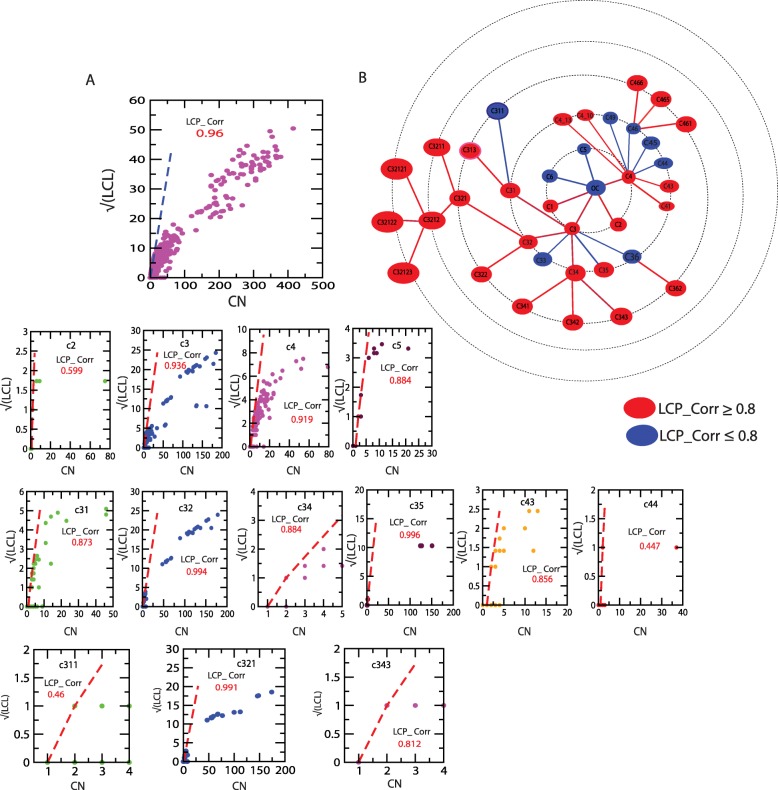
LCP correlation as a function of CN for different modules/sub-modules and their distribution

The compactness of the network/modules/sub-modules with size are calculated using LCP-DP algorithm which is expressed $\sqrt{LCL}$ (local community links) as a function of *CN* (common neighborhood) (see Methods) and found that the number of strongly connected networks/modules/sub-modules (*LCP* − *corr* ≥ 0.8) are greater than the number of loosely connected network/modules/sub-modules (*LCP* − *corr* < 0.8) at *s* = 1 (upper level of organization) (see Fig. 6). The size of the modules at *s* = 1 ranges from 10 to 180 nodes. However, as one moves from top to bottom (*s* > 1), the number of strongly connected modules decrease as compared to loosely connected modules/sub-modules. Since the ovarian cancer network is tightly bound at the upper level and complete network, the network itself is organized to maintain its own properties against any external and internal perturbations (both local and global) in the network.

Now the analysis of LCP-DP of the network/modules/sub-modules shows that, except one particular module and its corresponding sub-modules at different levels, all the other modules/sub-modules become loosely packed with decrease in size as one move from top to bottom levels. The particular module/sub-modules, whose size and compactness do not change much till third level (LCP-correlation ranges from 0.936 − 0.994 and size 175 − 180) is the module/sub-module in which all the five KRs are accommodated. This means that the module/sub-module is tightly regulated by these five KRs along with their connecting nodes in them (Fig. 6 second panel in each row). However, the removal of these five KRs do not cause network breakdown (Fig. 2). Hence, this module/sub-module still try to preserve its own properties against any local and global perturbations.

### Centrality lethality is ruled out in the ovarian cancer network

The ovarian cancer network obeys nearest to ideal hierarchical type of network, thus the emergent modules/sub-modules are tightly bound at top levels of organization. The removal of key regulators does not cause the network breakdown (Fig. 2). Even though one module in which the five KRs are accommodated and it is corresponding few sub-modules breakdown after the third level, other modules/sub-modules remain stable to protect the ovarian cancer network properties. So, ovarian cancer network lead out centrality-lethality rule [33]. But the identified key regulators have significant regulating activities in the ovarian cancer network that is reflected in the variations in the topological properties (Fig. 2) and another network parameter (Figs. 5 and 6) and its associated communities at different levels of organization.

### AKT1 plays central role in regulating ovarian cancer network

**Fig. 7:**
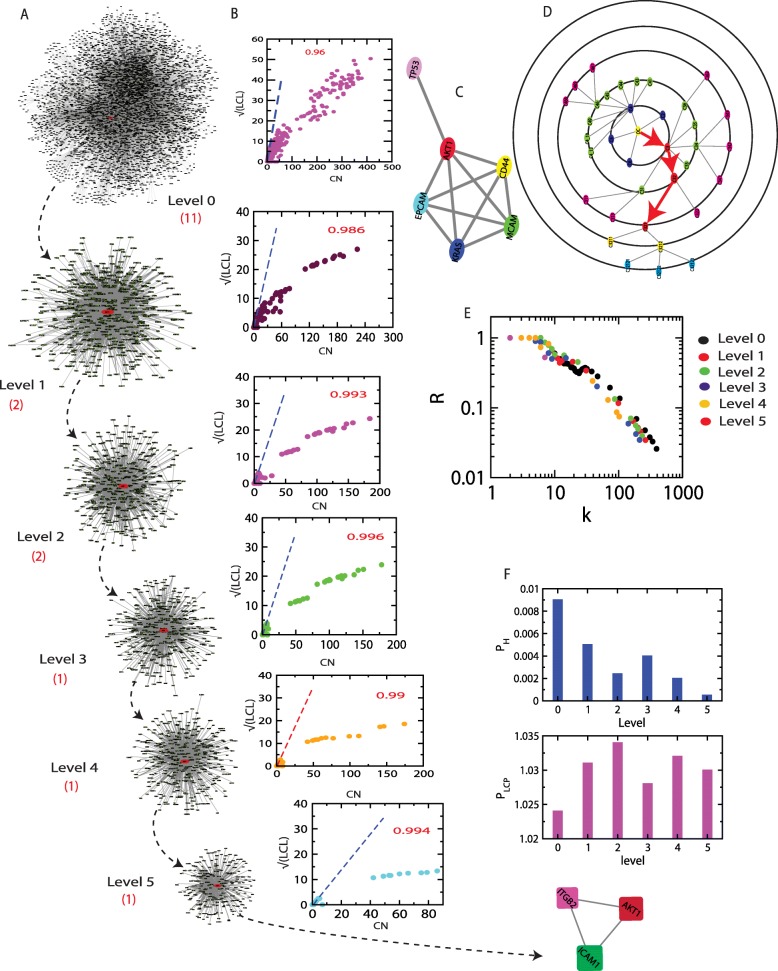
Properties of AKT1. **a**. The tracing of AKT1 in network/modules/sub-modules at different network levels. **b**. The variation of $\sqrt{LCL}$ as a function of CN for different levels. **c**. Organization of five key regulators with Tp53. **d**. Directional tracing of AKT1 at different network levels. Rich-club parameter as a function of k *P*_*H*_ and $\sqrt{LCP}$ as a function of level

AKT1, which is a modulator of apoptotic signal and important therapeutic target gene in ovarian cancer [34], is found to be tightly bound with other important leading ovarian cancer regulator genes with large extension of network/modular sizes 400 to 100 depending on the network level of organization indicated by LCP-DP calculations (see Methods, Fig. 7). In these calculations, the network/module/sub- module in which AKT1 is present are considered, where LCP-correlations of these networks/modules/sub-modules are found to be in the range [0.986 − 0.994] revealing strong compactness of these networks/module/sub-module at different levels of organization. Further, AKT1 is found to act as a main regulator which allows to crosstalk with other remaining KRs (CD44, MCAM, KRAS and EPCAM) and Tp53 (Fig. 7C). Since the clustering co-efficient of all five KRs in the extracting net- work of these five KRs are one (Fig. 7C), the identified five KRs are again found to be interacting strongly which is the signature of *rich-club* formation in the network [35]. However, if we consider the whole network, we could not able to capture the signature of this rich-club formation of these KRs as evident from network connectivity property dependence on negative exponent *C*_*N*_ (*k*) ∼ *k*^−*β*^ [29, 36] (Fig. 2A) and negative exponent dependence on rich-club parameter R on degree k, R ∼ k^−θ^ [35]. Since the rich-club data (R versus k) for all networks, module and sub-modules scale the same scaling functional dependence, *R* ∼ *k*^−*θ*^, the network organization exhibits absence of central controlling mechanism by AKT1 and its rich-club with KRs. Hence, even though AKT1 is significantly important KR in ovarian cancer network, it never tries to dominate the network organization at different levels of organization.

Now, to understand relative energy ATK1 can have at different levels of network organization, can be obtained as follows: Consider, *H*_*s*_ is the Hamiltonian function at any level of network organization s, where, *s* = 0, 1, …, 5 (network corresponding to *s* = 0 is the complete network). If *m*_*s*_ is the number of modules/sub-modules at level *s*, then Hamiltonian function per module/sub-module at level s is given by, ${H}_s=\frac{1}{m_s}\sum \limits_{j=1}^{m_s}\sum \limits_{c_j}\left({e}_{c_j}-{\mathrm{Yn}}_{c_j}^2\right)$, where ${n}_{c_j}$ is the size of the *j*^*th*^ module/sub-module at level *s*. Then the Hamiltonian function of AKT1 at the module it belongs to can be obtained as, ${H}^{\left[ AKT1\right]}=-\left({e}^{\left[ AKT1\right]}-{\mathrm{Yn}}_s^{\left[ AKT1\right]}\right)$, Where *e*^[*AKT*1]^ and ${\mathrm{n}}_s^{\left[ AKT1\right]}$ are the number of edges AKT1 has and the size of the module/sub-module where AKT1 belongs to respectively. Now the relative energy AKT1 can have at any level s can be obtained by,

8 $$ {U}_{AKT1}(s)=\frac{H_{AKT1}}{H_s}\sim {e}^{-{\phi}_s};{\forall}_s\in I $$

where, *ϕ* is a constant. This relative energy of AKT1, *U*_*AKT*1_ represents the energy associated with AKT1 constrained by the level of organization which could be related to the activities of AKT1 at different levels s. In an ovarian cancer network, the activity of AKT1 decreases as one goes down from top to bottom of the network (Fig. 7F) indicating its important regulating activity at complete network level than at a basic level.

Further, we calculate the relative compactness of the module/sub-module which accommodates ATK1 at different level s by using,

9 $$ {W}_{LCP}=\frac{L_{AKT1}}{\sum_{j=1}^{m_s}{L}_j};L\to LCP- corr{\forall}_s\in I $$

here, the sum is over non-zero LCP-correlations of the modules/sub-modules at each levels s. The estimated values of W_LCP (Fig. 7F) show that the relative compactness increases as one goes down from top to bottom level indicating the strong interaction of nodes at a lower level of organization.

### Ovarian cancer network exhibit active regulating mechanism of key regulators with modules

Since ovarian cancer network follows hierarchical network features, the emerged modules/sub-modules become important regulating units at different levels of the organization along with active participation of KRs in network phenotypes. The multi-functionality of the network could be the manifestation of the interacting emerged module/sub-modules at each network level to keep the network properties stable. The KRs could be important workers of integrating the components in each module/sub-module they belong to for efficient functioning, through optimal signal processing among the components organized by these KRs. The five identified KRs in fact form rich-club phenomena at each level of organization, however, the impact of this rich-club activity at each level is weak enough such that this perturbation is unable to cause a significant variation in the overall network topological properties. Besides the ovarian cancer network/modules/sub-modules are mostly tightly bound due to strong interaction among the nodes/genes. Hence, the removal of these important KRs do not cause network breakdown indicating absence of central control system, that is a signature of self-organization [37].

The network also exhibits topological properties close to the ideal hierarchical network indicated by equation (3) (Fig. 2) and therefore the regulating mechanism in the network is active (far from equilibrium) in order to maintain network properties. At the same time the topological properties of ovarian cancer network show power law nature that is suggesting the OC network obeys fractal behaviour. This fractal nature due to self-affine process in the network could be a signature of self-organization in the OC network [38].

The knock-out experiment of five key regulators from the original ovarian cancer network express that the altered properties in the OC network due to knock out of five KRs don’t cause significant variation in the network topology. This suggests that the system don’t adopt to vary by cause due to perturbation communicated by KRs knock-out from the ovarian cancer network. The network then reorganize itself and adapted to the transformed topological properties of ovarian cancer network. The ability to adapt to the change for a better network organization without breakdown of the system is another signature of self-organization in the network [39].

### Key regulators are correlated to disease progression in ovarian cancer

**Fig. 8:**
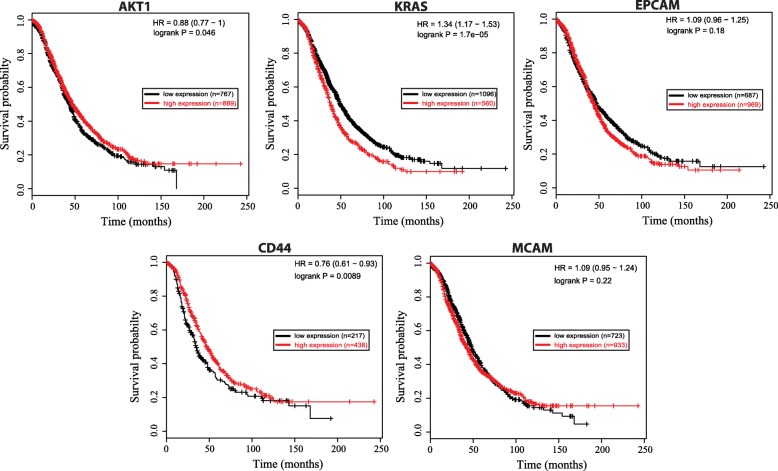
Kaplan-Meier curve of key regulators AKT1, KRAS, EPCAM, CD44 and MCAM. p-values were calculated using the log rank test to evaluate the overall survival analysis between low expression (black) and high expression (red) of key regulator genes of patients

Higher expressions of AKT1 and CD44 genes in ovarian cancer patients had higher probabilities of survival than their lower expressions (Fig. 8). This might be an indication that they act against the progression of the cancer and could play tumor suppressing roles. On the other hand, higher expressions of KRAS and EPCAM genes lower the probability of survival among OC patients thus their higher expressions could have tumorogenic effects and related to disease progression. But in case of MCAM gene, higher or lower expressions had equal impact on the overall survival of the patients (Fig. 8). Hence, expressions of key regulators AKT1, KRAS, EPCAM and CD44 can be correlated to increasing or decreasing the risk of disease progression, so, they can be potential prognostics markers or drug targets in ovarian cancer.

### Gene ontology (GO) and pathways analysis of community at the level 5 module

We have analysed pathways of level 5. The most significant pathways associated are given in Table 1. The p-value denotes the statistical significance of the pathways in the network. The other associated pathways, which are also statistically significant, such as RAS signalling, leucocytes trans endothelial migration, thyroid signalling pathways etc are listed in Table 1. These pathways are also been reported in many cancer types.

## Discussion

Complex ovarian regulatory network generated from experimentally verified set of genes show hierarchical features, which allows the genes to organize in a few different pathways (modules/sub-modules) in a complicated way, to exhibit multi- functionality of the system. Since the network bears hierarchical properties, the activities of individual gene are not much important, but their co-ordination exhibit different important functional special deeds. At the same time, some of the leading hubs in the network have significantly important functions, for example, integration of large number of lower degree nodes in the network for organizing and regulating activities, serve as a means of intra and inter cross-talk among different other essential genes, maintaining network inherent properties, stability and optimizing signal processing in the network. Though, out of these leading hubs, few hubs, which we term as *key regulator* s, acquire significantly more important roles in keeping network properties in better perspectives (ability to get adaptation to the fit change) [40]. In ovarian cancer network, out of seventy leading hubs in the ovarian cancer network, we could able to explore five such KRs which are AKT1, CD44, MCAM, KRAS and EPCAM. These KRs are deeply rooted in the network, they act as the backbone of the network for any network regulations and activities and could be a possible target gene for this disease control mechanisms. Surprisingly, the first few most popular hubs (eleven hubs) do not fall in KRs and these KRs need not necessarily be most popular hubs, but some of them keep a low profile in the network. These KRs form tightly bound rich-club, but the regulating activity of this rich-club could not able to show up in the network properties because the number of members in the rich-club is negligibly small as compared to the whole network. Further, these five KRs fall in a single module/sub-module up to fourth level of organization indicating closer working of the KRs and then start separating afterwards. Some of these identified KRs have experimentally been shown important backbone genes in ovarian cancer. For example, AKT1 is experimentally found therapeutic target gene [34], CD44 is found to be target gene, which serve as the backbone for paclitaxel prodrugs [41], MCAM is reported to be an important metastasis marker and invasion of ovarian cancer cells [42], KRAS is identified as important genetic marker of ovarian cancer [43]. However, even though EPCAM is involved in ovarian cancer regulation [44], we propose that this EPCAM could be an important gene for possible target gene in ovarian cancer.

Since the ovarian cancer network bears hierarchical properties, removal of these KRs does not cause network breakdown, rather reorganize the network to an- other perspective and adapt to it. Since the five KRs are associated with a single module/sub-module, one can target (possible drug target genes) these KRs and accommodating module/sub-module in ovarian cancer. But removal of KRs from the module they belong to cause modular breakdown after a certain level of organization in the network. Hence, one needs to investigate this module/sub-module for the critical target of this disease.

Higher and lower expression of the key regulatory genes can be correlated with progression of tumorogenesis and overall survival among ovarian cancer patients. In addition to the topological properties of the modules of the protein-protein interaction network of ovarian cancer, the predicted important pathways from the network modules are found to be associated with different other types of cancer.

## Conclusions

This study proposes a new method to identify key regulators of ovarian cancer net- work. The ovarian cancer network is a tightly bound network and follows certain properties: first, the network rules out centrality-lethality rule (no central control system); second, network topology obeys fractal laws; third, out of KRs AKT1 plays central role in regulating ovarian cancer system. However, we need to study large scale analysis of the dynamical network, which involves different biologically well-defined modules to understand the time evolution of the ovarian cancer and for spatio-temporal behaviors of target genes.

## Supplementary information

**Additional file 1.** Supplementary file of methods.

## Data Availability

The data used in the current study available from the corresponding author on reasonable request.

## Abbreviations

C(k): Clustering co-efficient
C_B_: Betweenness
C_C_: Closeness
C_E_: Eigen-value centrality
C_N_ (k): Neighborhood connectivity
CN: Common neighborhood
DAVID: Database for Annotation, Visualization and Integrated Discovery
EOC: Epithelial ovarian cancers
GO: Gene ontology
H_s_: Hamiltonian function
KRs: Key regulators
LCL: Local community links
LCP-DP: Local community paradigm decomposition plot
OC: Ovarian cancer
p(k): Degree distribution
PPI: Protein-protein interaction
TCGA: The Cancer Genome Atlas
TF: Transcriptional factor
