# Supplementary material for: Methodology of predicting novel key regulators in ovarian cancer network: a network theoretical approach
Source: BMC Cancer. 2019 Nov 21;19:1129. doi: 10.1186/s12885-019-6309-6 (PMC6869253; doi:10.1186/s12885-019-6309-6)
Supplement: Supplementary file 1 — Additional file 1. Supplementary file of methods. [file 12885_2019_6309_MOESM1_ESM.docx]

**Supplementary file on**

**Methodology of predicting key regulators in ovarian cancer network: A network theoretical approach**

Md Zubbair Malik[^†^](#_bookmark0), Keilash Chirom^3^[^†^](#_bookmark0), Shahnawaz Ali^2^, Romana Ishrat^2^,

Pallavi Somvanshi^3^ and R.K. Brojen Singh^1^[^*^](#_bookmark0)

1*School of Computational* & *Integrative Sciences, Jawaharlal Nehru University, New Delhi-110067, India.*

2 *Centre for Interdisciplinary Research in Basic Sciences, Jamia Millia Islamia, New Delhi-110025, India.*

3*Department of Biotechnology, TERI University, New Delhi, India.*

∗ Authors have equal contributions

^[†](#_bookmark0)^ Electronic address: R. K. Brojen Singh: [brojen@jnu.ac.in](mailto:brojen@jnu.ac.in) (Corresponding author)

**Methods**

**Acquisition of ovarian cancer data**

We have integrated 6 hugely cited resources for cancer in procedure to access the gene of ovarian cancer. The various resources focus on various aspects of cancer biology: (1) COSMIC database: it stores mutated genes information causing cancer, (2) Gene cards database: It provides information of human genes related to their genetic and functional information (3) Ovarian kaleidoscope database: It provides information regarding the biological function, expression pattern, and regulation of genes expressed in the ovary and their mutation. (4) Dragon database of ovarian cancer: It stores functional information on genes in ovarian cancer. (5) Curated ovarian database: it contains manually curated data for gene expression from meta-analysis of patients suffering from ovarian cancer. (6) OCGene database contains the list of experimentally verified ovarian cancer-related genes.

We have got 2000 genes from the above assimilated repositories. We followed the protocol in this procedure is a simple workflow focus along the mining of ovarian cancer associated gene list from all of the 6 described databases. The above-mentioned lists were then incorporated to CGI code written in Perl to remove duplication genes which were redundant in names as well as aliases used for names of the gene. The process of removal suggests searching globally and pattern matching in Gene card database (http://www.genecards.com). This procedure allows us to retrieve the information of unique 660 genes along with their synonymic names (in csv-format). Now, the extracted gene list is put into the manual curation with reference to the agilent literature (plugin of Cytoscape) search, to obtain the associated literature of each gene obtained. At last, we could able to arrive at the final list of 600 genes after curation from the 2000 unique genes. Now, we mapped these curated 600 genes to UniProt (June 2018) to retrieve names, UniProt-ID and other related information. After collecting the required in- formation of the curated 600 genes, we constructed primary ovarian cancer network using these genes for further study.

**Construction of ovarian cancer PPI network**

We followed the simple concept of one gene one protein correspondence and constructed the primary ovarian cancer Protein-Protein Interaction (PPI) network from the 600 curated genes. The network was constructed using GeneMANIA database [[1](#_bookmark1)] and verified and uploaded the file in Cytoscape [[2](#_bookmark2)] for further literature verification. We used UniProt, OCgene database verification; this analytical and integrative effort done provided an efficient way to curate and verify experimentally, the PPIs. Within the framework of network theory, this complex OC network can be represented by a graph $G=(N, E)$, where, $E = e_{ij} ; i, j = 1, 2, . . . ,$ *N* is the set of edges assigned among any pair of nodes in the network, and $N = n_{i} ; i = 1, 2, . . . , N$ is the set of nodes in the network. We then cross-checked the network with five well studied driver genes AKT1, KRAS, EPCAM, CD44 and MCAM, after using the InACT database thousands of interaction were obtained. After the integrating all the required information and data curation, we finally obtained an ovarian cancer network of $4818$ nodes having $16320$ connections in the constructed network.

**Topological analyses of the networks**

The characteristics of the network structural properties can be well studied by analyzing the nature of network parameters given in the following [[3](#_bookmark3), [4](#_bookmark4)].

**Degree**

In the analysis of network, degree $k$indicates the total number of links established by a node in a network and is used to measure the local significance of a node in

regulating the network. In a graph represented by $G= (N, E),$ where $N$ and $E$ are sets of nodes and edges of a network respectively, the degree of $i^{th}$ node ($k$) is expressed as $k_{i}=\sum_{ij}^{N} A_{ij}$, where $A_{ij}$ denotes the adjacency matrix elements of the graph.

**Degree distribution**

It is the probability of a random node to have a degree out of the total number of nodes in the network and is represented as fraction of nodes having degree as shown in Equation (1), where $N_{k}$ is the total number of nodes with degree ($k$) and total nodes in the network.

$P\left( k \right)=\frac{n_{k}}{N}$ (1)

*P* (*k*) of random and small-world networks follow Poisson distribution [[5](#_bookmark5)] in degree

distribution against degree, however, most of the real-world networks, which are generally scale-free and hierarchical networks obey power law nature $P \left( k \right)\sim k^{-\gamma}$ [5, [6](#_bookmark6)], where, $4\geq\gamma\leq2$ . In hierarchical networks $\gamma\sim2.26$, (mean-field value) indicating a modular organization at different topological levels [[7](#_bookmark7), [8](#_bookmark8)]. Therefore, the exhibited patterns define the characteristic topology of a network.

**Clustering co-efficient**

The strength of internal connectivity among the nodes neighbourhoods which quantifies the inherent clustering tendency of the nodes in the network is characterized by the Clustering coefficient $C(k)$ which is the ratio of the number of triangular motifs formed by a node with its nearest neighbors to the maximum possible number of triangular motifs in the network. For any node $i$ having degree $k_{i}$in an undirected graph, $C(k)$ can be expressed as,

$C\left( k_{i} \right)=\frac{2e_{i}}{k_{i}(k_{i}-1)}$ (2)

where *e_i_* is the total number of edges among its nearest neighbours. In scale-free networks, one can find this nature, $C(k) \sim constant$, but it exhibits power law in hierarchical network against degree, $C(k) \sim k^{-\alpha,}$ where, $\alpha\sim1$ [[7](#_bookmark7)–[9](#_bookmark9)].

**Neighborhood connectivity**

The node neighborhood connectivity ($C_{N}(k)$) average connectivity established by the nearest neighbours of a node with degree $k$, represented by $C_{N}\left( k \right)$ can be expressed as shown in Equation (3),

$C_{N} \left( k \right)=\sum_{q} qP (q|k)$ (3)

where, $P(q|k)$ is conditional probability of the links of a node with $k$ connections to another node having $q$ connections. In hierarchical network topology, *C_N_* (*k*) exhibit power law against degree $k$ that is, $C_{N}\left( k \right)\sim k^{\beta}$, where, $\beta\sim0.5$ [[11](#_bookmark10)]. Further, the positivity or negativity of the exponent $\beta$ can be defined as, respectively, the assortivity or disassortivity nature of a network topology [[12](#_bookmark11)].

**Betweenness centrality**

Betweenness Centrality $(CB\left( v \right))$ is the measure of a node which is the share of all shortest-path traffic from all possible routes through nodes $i$ to $j$. Thus, it characterizes a node’s ability to benefit from the propagation of information in the network [[13](#_bookmark12)] and its controlling ability of signal processing over other remaining nodes in the network [[14](#_bookmark13), [15](#_bookmark14)]. If $d_{ij} (v)$ denotes the number of geodesic paths connecting node $i$ to node $j$ passing through node *v*, then $C_{B}(v)$ can be measured by,

$$C_{b}(v)=\sum_{i,j;i\neq j\neq k} \frac{d_{ij}(\nu)}{d_{ij}}$$

(4)

Now, if *M* is the number of pairs of nodes excluding *v*, one can define normalized betweenness centrality by the following equation,

$C_{B}(v) =\frac{1}{M}(C_{b}(v)$ (5)

**Closeness centrality**

The closeness centrality ${(C}_{C})$represents the total geodesic distance from a given node to all its other connected nodes. It represents the speed of spreading of information in a network from a node to other connected nodes [16]. $C_{C}$of a node $i$ in a network is calculated by the division of total number of nodes in the network, n by sum of geodesic path lengths between nodes $i$and $j$ which is represented by $d_{ij}$ in Equation (6).

$$C_{C} \left( k \right)=\frac{n}{\sum_{j} d_{ij}} (6)$$

**Eigenvector centrality**

Eigenvector centrality$C_{E}$ is proportional to the sum of the centrality of all neighbours of a node and it reflects the intensity of these most prominent nodes influencing the signal processing in the network [17]. If nearest neighbours of node $i$ in the network is denoted by $nn(i)$ with eigenvalue $\lambda$ and eigenvector $v_{i}$ of eigen-value equations, $Av_{i}=\lambda v_{i}$where, $A$ is the network adjacency matrix, $C_{E}$ can be shown by the Equation (7),

$$C_{E} \left( i \right)=\frac{1}{\lambda}\sum_{j=nn(i)} v_{j} (7)$$

$C_{E}$ score provides $\lambda_{max}$, which is the maximum positive eigenvalue of the principal eigenvector of $A$ [18]. Since a node $C_{E}$ function depends on the centralities of its neighbours, it varies across different networks association of high $C_{E}$ nodes; within closely connected locality of such nodes reduces the chances of isolation of nodes [17]. Thus, $C_{E}$becomes a powerful indicator of information transmission power of a node in the network.

**Tracking of genes and Knock out experiment**

The most influential genes in the OC network were identified first through calculating the centrality measures. Since, higher degree nodes have higher centrality values, top 70 highest degree nodes were considered among the hub nodes of the network for tracing the key regulators which may play important role in regulating the network. Then tracing of nodes from the primary network up to motif level was done on the basis of representation of the respective nodes (proteins) across the sub modules obtained from Newman and Grivan’s method of community detection/clustering. Finally, the hub-nodes (proteins) which were represented at the modules at every hierarchical level were considered as key regulators of the OC network. Then knockout experiment of leading hubs including these key genes were performed to understand the change in the network properties in the absence of the leading hubs. We successively removed the leading hub genes from the generated primary OC network, after that we calculated various topological properties of the modified network to study the regulating capabilities of the hub genes and their importance. We also studied the changes in the topological properties of various sub-networks at different levels of network organization to understand the regulating roles of these genes even at various levels of the network. We used Network analyzer plugin in Cytoscape version 3.7.1 to calculate various network parameters. However, for eigen value estimation we used CytoNCA (plugin in Cytoscape). The results obtained from these two plugins were cross checked.

**Method for community detection: Leading eigen-vector method**

There is no specific definition of community, however, it can be defined as the densely connected group of nodes which can probably able to perform certain specific function. The modular structure of a complex network, which generally have hierarchical levels of organization, can be detected using leading eigen-vector method. These detected communities may have different functionalities, properties and regulating roles. There have been proposed various algorithms for the detection of communities in a network, out of which leading eigen-vector method (LEV) provide a promisingly accurate results (in our case), because the algorithm measures the eigenvalue for each link, giving importance to links not to nodes. Hence, we used LEV detection method in R using the $igraph$ package. This method was used in our network analysis to detect communities/sub-communities from OC network at various levels of organization and continued this process until the level of motifs (i.e. 3 nodes and 3 edges). In the whole process, we sticked to the criterion of identifying last level of organization, where, any one of the sub-modules have at least one motif (defined by $G(3, 3).$

**Hamiltonian energy calculation: Distribution of energy in the network**

The energy associated with a network at a certain level can be calculated from the Hamiltonian energy ($HE$) function of the network at that level within the framework of Constant Potts Model [20, 21]. This $HE$ of the network is contributed from the energy distributions of modules/sub-modules at various levels of organization, as well as, intermodular interaction. Hence, the $HE$ of a network or module or sub-module can be measured by,

$$H^{c}=-\sum_{c} \left[ e_{c}-{\Upsilon n}_{c}^{2} \right] (8)$$

where $e_{c}$ and $n_{c}$ are the number of edges and nodes in a community $‘c’$ and is the resolution parameter acting as an edge density threshold. Generally, one can have, $\Upsilon\leq\frac{1}{\left( n_{c} \right)^{2}}$.

**Method for charactering network compactness: LCP − DP approach**

The local community paradigm-decomposition-plot $(LCP -DP)$ method is one way of characterizing the compactness of a network by representing network properties in $2D$ space of common neighbors $(CN)$ index of interacting nodes and local community links $(LCL)$ of each pair of interacting nodes in the network. The method could provide us information of number, size, and compactness of a network, as well as, the modules in the network, which can be used as an order parameter to characterize the properties of self-organization in the network [19]. The $CN$index between two nodes $x$ and $y$ in a network can be calculated from the amount of overlap between their sets of first node-neighbors $S(x)$ and $S(y)$, which is denoted by,

$CN = S\left( x \right) \bigcap S(y)$. From this method, one can qualify the interaction of any two nodes in the network if $S\left( x \right) \bigcap S(y)$. has significantly large number (i.e. having significant amount of overlapping). Larger the values of $CN$ correspond to the increase in compactness in the network, which could be the indication of faster signal processing in the network. On the other hand, the $LCL$ between the two nodes $x$ and$y$, whose upper bound is defined by,

$\max(LCL) =\frac{1}{2}(CN-1)$, is the number of internal links in the local community ($LC$). The two nodes are likely to be linked together if $CN$ of these two nodes are members of $LC$

[19]. In $LCP - DP$ plot, $CN$ has linear dependence with $\sqrt{LCL}$.

The $LCP$ correlation ($LCP -corr$) is the Pearson correlation co-efficient of $CN$ and $LCL$defined by $LCP -corr=\frac{cov(CN,LCL)}{\sigma_{CN} \sigma_{LCL}}$ with $CN>1,$ where $cov(CN,LCL)$ is the covariance between $CN$ and $LCL$, $\sigma_{CN}$and $\sigma_{LCL}$ are standard deviations of $CN$ and $LCL$, respectively.

**References**

1. Sara M, Ray D, Farley DW, Grouios C & Morris Q. GeneMANIA: a real-time multiple association network integration algorithm for predicting gene function. Genome biology. 2008; 9(1);S4.
2. Paul S, Markiel A, Ozier O, Baliga NS, Jonathan T. Wang, Daniel R, Nada A, Schwikowski B & Ideker T. Cytoscape: a software environment for integrated models of biomolecular interaction networks. Genome research. 2003;13(11);2498-2504.
3. Ali S, Malik MZ, Singh SS, Chirom K, Ishrat I & Singh RKB. Exploring novel key regulators in breast cancer network. PloS one. 2018;13(6); e0198525.
4. Anam F, Tazyeen S, Ahmed MM, Alam A, Ali S, Malik MZ, Ali S & Romana I. Assessment of the key regulatory genes and their Interologs for Turner Syndrome employing network approach. Scientific reports. 2018; 8(1);10091.
5. Barabsi AL & Albert R. Emergence of scaling in random networks. science. 1999;286(5439);509-512.
6. Albert R & Barabsi AL. Statistical mechanics of complex networks. Reviews of modern physics. 2002;74(1);47-97.
7. Ravasz E, Somera AL,Mongru DA, Oltvai ZN & Barabsi AL. Hierarchical organization of modularity in metabolic networks. science. 2002;297(5586); 1551-1555.
8. Ravasz E, et. al. Hierarchical organization in complex networks. Phys. Rev., E. 2003;67;026112.
9. Barabasi AL & Oltvai ZN. Network biology: understanding the cell’s functional organization. Nature reviews genetics. 2004;5(2);101-113.
10. Maslov S & Sneppen K. Specificity and stability in topology of protein networks. Science. 2002;296(5569);910-913.
11. Pastor-Satorras R, Vzquez A & Vespignani A. Dynamical and correlation properties of the Internet. Physical review letters. 2001;87(25), 258701.
12. Barrat A, Barthelemy M, Pastor-Satorras R & Vespignani A. The architecture of complex weighted networks. PNAS, USA. 2004;101(11);3747-3752.
13. Borgatti SP & Everett MG. A graph-theoretic perspective on centrality. Social networks. 2006;28(4);466-484.
14. Brandes U. A faster algorithm for betweenness centrality. J. Math. Sociol. 2001;25;163-177.
15. Mason O & Verwoerd M. Graph theory and networks in biology. IET systems biology. 2007;1(2);89-119.
16. Canright G & Engo-Monsen K. Roles in networks. Science of Computer Programming. 2004;53(2);195-214.
17. Canright GS & Engo-Monsen K. Spreading on networks: a topographic view. Complexus. 2006;3(1-3);131-146.
18. Bonacich P. Power and centrality: A family of measures. American journal of sociology. 1987;1170-1182.
19. Cannistraci CV, Alanis-Lobato G & Ravasi T. From link-prediction in brain connectomes and protein interactomes to the local-community-paradigm in complex networks. Scientific reports. 2013;3;1613.
20. Traag VA, Van Dooren P & Nesterov Y. Narrow scope for resolution-limit-free community detection. Physical Review E. 2011;84(1);016114.
21. Traag VA, Krings G & Van Dooren P. Significant scales in community structure. Scientific Report. 2013;3;2930.
22. Liu Y, Xia J, Sun J & Zhao M. OCGene: a database of experimentally verified ovarian cancer-related genes with precomputed regulation information. Cell death & disease. 2015;6(12);e2036.
23. Clauset A, Shalizi CR & Newman ME. Power-law distributions in empirical data. SIAM review. 2009;51(4);661-703.
24. Mandelbrot B, Fisher A & Calvet L. A multifractal model of asset returns. Cowles Foundation Discussion. 1997; Paper No.1164.
25. Mandelbrot BB. Negative fractal dimensions and multifractals. Physica A: Statistical Mechanics and its Applications.1990 163(1), 306-315.
26. Newman ME & Girvan M. Finding and evaluating community structure in networks. Physical review E. 2004;69(2);026113.
27. Altomare DA, Wang HQ, Skele KL, De Rienzo A, Klein-Szanto AJ, Godwin AK & Testa JR. AKT and mTOR phosphorylation is frequently detected in ovarian cancer and can be targeted to disrupt ovarian tumor cell growth. Oncogene. 23(34); 5853-5857.
28. Colizza V, Flammini A, Serrano MA, & Vespignani A. Detecting rich-club ordering in complex networks. Nature physics. 2006;2(2);110-115.
29. Kauffman SA. The origins of order: Self organization and selection in evolution. Oxford University Press, USA. 1993.
30. Heylighen F. The science of self-organization and adaptivity. The encyclopedia of life support systems. 2001;5(3);253-280.
31. Ashby WR. Principles of the self-organizing system. In Facets of Systems Science. Springer US. 1991;521-536).
32. Auzenne E, Ghosh SC, Khodadadian M, Rivera B, Farquhar D, Price RE & Klostergaard J. Hyaluronic acid-paclitaxel: antitumor efficacy against CD44 (+) human ovarian carcinoma xenografts. Neoplasia. 2007;9(6);479-486.
33. Wu Z, Wu Z, Li J, Yang X, Wang Y, Yu Y & Zhang Z. MCAM is a novel metastasis marker and regulates spreading, apoptosis and invasion of ovarian cancer cells. 2012;Tumor Biology;33(5);1619-1628.
34. Ratner E, Lu L, Boeke M, Barnett R, Nallur S, Chin LJ & Hui P. A KRAS-variant in ovarian cancer acts as a genetic marker of cancer risk. Cancer research. 2010;70(16);6509-6515.
35. Baeuerle PA & Gires O. EpCAM (CD326) finding its role in cancer. British journal of cancer. 2007;96(3);417-423.

**Table S1:** List of experimentally verified genes involved in ovarian cancer which used for our network construction, with their description and functions in the cancer network

| S.No | | Gene  symbol | | Gene id | | Description | Locus | | Function | | HGNCid | | Reference | |  |
| --- | --- | --- | --- | --- | --- | --- | --- | --- | --- | --- | --- | --- | --- | --- | --- |
| 1 | | ATM | | 472 | | Ataxia telangiectasia mutated | 11q22-q23 | | cell cycle regulation, DNA Repair | | 795 | | (22) | |  |
| 2 | | **MSH3** | | 4437 | | MutS homolog 3 (E.coli) | 5q11-q12 | | DNA mismatch repair | | 7326 | | (22) | |  |
| 3 | | **MSH6** | | 2956 | | MutS homolog 6 (E.coli) | 2p16 | | Recognition of mismatched re-  pair | | 7329 | | (22) | |  |
| 4 | | MYC | | 4609 | | V-myc avian myelocy- tomatosis viral oncogene homolog | 8q24.21 | | Regulates transcription | | 7553 | | (22) | |  |
| 5 | | CDH1 | | 999 | | Cadherin 1, type 1, Ecadherin (epithelial) | 16q22.1 | | Protein encodeing | | 1748 | | (22) | |  |
| 6 | | ATR | | 545 | | ATR serine/threonine Kinase | 3q23 | | cell cycle regulation, DNA Re- pair | | 882 | | (22) | |  |
| 7 | | MLH1 | | 4292 | | MutL homolog 1 | 3p21.3 | | Chromosome segregation, DNA repair | | 7127 | | (22) | |  |
| 8 | | NBN | | 4683 | | Nibrin | 8q21 | | DNA damage-induced checkpoint activation | | 7652 | | (22) | |  |
| 9 | | STAT3 | | 6774 | | Signal transducer and activator of transcription 3 (acute-phase response fac-  tor) | 17q21.31 | | Transcription activator | | 11364 | | (22) | |  |
| 10 | | TP53 | | 7157 | | Tumor protein p53 | 17p13.1 | | Regulate expression of target genes | | 11998 | | (22) | |  |
| 11 | | AURKA | | 6790 | | Aurora kinase A | 20q13 | | Microtubule formation and/or stabilization during chromo-some segregation | | 11393 | | (22) | |  |
| 12 | | **AKT1** | | 207 | | v-akt murine thymoma viral oncogene homolog 1 | 14q32.32 | | Induced Cell death, Cell differentiation | | 391 | | (22) | |  |
| 13 | | AXIN2 | | 8313 | | Axis Inhibition Protein 2 | 17q24.1 | | Regulation of Wnt-mediated beta catenin signaling | | 904 | | (22) | |  |
| 14 | | NF1 | | 4763 | | Neurofibromin 1 | 17q11.2 | | Negative regulator of the RAS signal transduction pathway | | 7765 | | (22) | |  |
| 15 | | ERCC1 | | 2067 | | Excision repair cross- complementation group 1 | 19q13.32 | | Repair of DNA lesions. | | 3433 | | (22) | |  |
| 16 | | MAP2K4 | | 6416 | | Mitogen-activated protein kinase kinase 4 | 17p12 | | proliferation, differentiation, transcription regulation, and development. | | 6844 | | (22) | |  |
| 17 | | FN1 | | 2335 | | Fibronectin 1 | 2q34 | | stem cell differentiation | | 3778 | | (22) | |  |
| 18 | | FANCD2 | | 2177 | | Fanconi anemia, comple- mentation group D2 | 3p26 | | homology-directed DNA repair, splicing | | 3585 | | (22) | |  |
| 19 | | MVP | | 9961 | | Major vault protein | 16p11.2 | | Multidrug resistance | | 7531 | | (22) | |  |
| 20 | | MAP3K1 | | 4214 | | Mitogen-activated protein kinase 1, E3 ubiquitin protein ligase | 5q11.2 | | Phosphorylation | | 6848 | | (22) | |  |
| 21 | | MAPK1 | | 5594 | | Mitogen-activated protein kinase 1 | 22q11.21 | | Proliferation, differentiation, transcription regulation and development. | | 6871 | | (22) | |  |
| 22. | | ESR1 | | 2099 | | Estrogen receptor 1 | 6q25.1 | | DNA binding, and activation of transcription. | | 3467 | | (22) | |  |
| 23 | | ERBB3 | | 2065 | | Erb-b2 receptor tyrosine ki- nase 3 | 12q13 | | prognostic prostate, bladder, and breast tumors. | | 3431 | | (22) | |  |
| 24 | | ABCB1 | | 5243 | | ATP-binding cassette, sub- family B (MDR/TAP), member 1 | 7q21.12 | | Multidrug-resistant cells and transporter | | 40 | | (22) | |  |
| 25 | | KRAS | | 3845 | | Kirsten rat sarcoma viral oncogene homolog | 12p12.1 | | cancer | | 6407 | | (22) | |  |
| 26 | | AR | | 367 | | Androgen receptor | Xq12 | | Transcription of androgen responsive genes | | 644 | | (22) | |  |
| 27 | | EPCAM | | 4072 | | Epithelial cell adhesion molecule | 2p21 | | Congenital tufting enteropathy | | 11529 | | (22) | |  |
| 28 | SMAD4 | | 4089 | | SMAD family member 4 | | | 18q21.1 | | Signal transduction proteins | | 6770 | | (22) | |
| 29 | PIK3CA | | 5290 | | Phosphatidylinositol-4,5- bisphosphate 3-kinase, catalytic subunit alpha | | | 3q26.3 | | oncogenic | | 8975 | | (22) | |
| 30 | BRCA1 | | 672 | | Breast cancer 1, early onset | | | 17q21 | | Maintaining genomic stability, and tumor suppression | | 1100 | | (22) | |
| 31 | RB1 | | 5925 | | Retinoblastoma 1 | | | 13q14.2 | | Negative regulator of the cell cy- cle | | 9884 | | (22) | |
| 32 | MDM4 | | 4194 | | MDM4, p53 regulator | | | 1q32 | | p53 transactivation and apop- totic functions | | 6974 | | (22) | |
| 33 | BARD1 | | 580 | | BRCA1 associated RING domain 1 | | | 2q35 | | DNA repair and/or transcrip- tional activation | | 952 | | (22) | |
| 34 | GSTP1 | | 2950 | | Glutathione S-transferase pi 1 | | | 11q13 | | Detoxification, xenobiotic metabolism and susceptibility to cancer | | 4638 | | (22) | |
| 35 | CDK2 | | 1017 | | Cyclin-dependent kinase 2 | | | 12q13 | | Cell cycle regulation. Activity of this protein is especially critical during the G1 to S phase transition. | | 1771 | | (22) | |
| 36 | ERBB2 | | 2064 | | Erb-b2 receptor tyrosine ki- nase 2 | | | 17q12 | | This gene encodes a member of the epidermal growth factor (EGF) receptor family of receptor tyrosine kinases | | 3430 | | (22) | |
| 37 | NCOA3 | | 8202 | | Nuclear receptor coactivator 3 | | | 20q12 | | Regulates transcriptional activator functions, complex fromation | | 7670 | | (22) | |
| 38 | APC | | 324 | | Adenomatous polyposis coli | | | 5q21-q22 | | cell migration and adhesion, transcriptional activation, and apoptosis | | 583 | | (22) | |
| 39 | ITGB3 | | 3690 | | Integrin, beta 3 (platelet glycoprotein IIIa, antigen CD61) | | | 17q21.32 | | cell adhesion and cell-surface : mediated signalling | | 6156 | | (22) | |
| 40 | RAD51 | | 5888 | | Recombinase | | | 15q15.1 | | homologous recombination and repair of DNA. | | 9817 | | (22) | |
| 41 | CD44 | | 960 | | CD44 molecule (Indian blood group) | | | 11p13 | | cell-cell interactions, cell adhe- sion and migration | | 1681 | | (22) | |
| 42 | HRAS | | 3265 | | Harvey rat sarcoma viral oncogene homolog | | | 11p15.5 | | implicated in a variety of cancer | | 5173 | | (22) | |
| 43 | DNMT1 | | 1786 | | DNA (cytosine-5-)- methyltransferase 1 | | | 19p13.2 | | methylation patterns following DNA replication | | 2976 | | (22) | |
| 44 | BIRC5 | | 332 | | Baculoviral IAP repeat con- taining 5 | | | 17q25 | | Inhibitor of apoptosis (IAP) | | 593 | | (22) | |
| 45 | CDKN1A | | 1026 | | Cyclin-dependent kinase in- hibitor 1A (p21, Cip1) | | | 6p21.2 | | Regulator of cell cycle progres- sion at G1 | | 1784 | | (22) | |
| 46 | BCL2 | | 596 | | B-cell CLL/lymphoma 2 | | | 18q21.3 | | Blocks the apoptotic death. | | 990 | | (22) | |
| 47 | MCAM | | 4162 | | Melanoma cell adhesion molecule | | | 11q23.3 | |  | | 6934 | | (22) | |
| 48 | ERCC3 | | 2071 | | Excision repair cross- complementation group 3 | | | 2q21 | | Nucleotide excision repair | | 3435 | | (22) | |
| 49 | CCND1 | | 595 | | Cyclin D1 | | | 11q13 | | CDK kinases | | 1582 | | (22) | |
| 50 | E2F2 | | 1870 | | Transcription factor 2 | | | 1p36 | | cell cycle regulation | | 3114 | | (22) | |
| 51 | HIF1A | | 3091 | | Hypoxia inducible factor 1, alpha subunit (basic helix- loop-helix transcription fac- tor) | | | 14q23.2 | | Master regulator of cellular and systemic homeostatic response  to hypoxia | | 4910 | | (22) | |
| 52 | CDK4 | | 1019 | | Cyclin-dependent kinase 4 | | | 12q14 | |  | |  | | (22) | |
| 53 | PARK2 | | 5071 | | Parkin RBR E3 ubiquitin  protein ligase | | | 6q25.2-q27 | | The precise function of this gene  is unknown, involved in parkin- son. | | 8607 | | (22) | |
| 54 | MKI67 | | 4288 | | Marker of proliferation Ki- 67 | | | 10q26.2 | | Cellular proliferation | | 7107 | | (22) | |
| 55 | SOD2 | | 6648 | | Superoxide dismutase 2, mitochondrial | | | 6q25.3 | | Associated with idiopathic car- diomyopathy (IDC), premature  aging, sporadic motor neuron disease, and cancer | | 11180 | | (22) | |
| 56 | TERT | | 7015 | | Telomerase reverse tran- scriptase | | | 5p15.33 | | Cellular senescence | | 11730 | | (22) | |
| 57 | INSR | | 3643 | | Insulin receptor | | | 19p13.3- p13.2 | | Receptor tyrosine kinase which mediates the pleiotropic actions  of insulin | | 6091 | | (22) | |
| 58 | IGF1R | | 3480 | | Insulin-like growth factor 1 receptor | | | 15q26.3 | | Anti-apoptotic agent by enhancing cell survival. | | 5465 | | (22) | |
| 59 | CDKN1B | | 1027 | | Cyclin-dependent kinase inhibitor 1B (p27, Kip1) | | | 12p13.1-  p12 | | Cell cycle progression at G1 | | 1785 | | (22) | |
| 60 | BCL2L1 | | 598 | | BCL2-like 1 | | | 20q11.21 | | Anti- or pro-apoptotic regulation, Mitochondrial membrane channel (VDAC) opening | | 992 | | (22) | |
| 61 | BRCA2 | | 675 | | Breast cancer 2, early onset | | | 13q12.3 | | Maintenance of genome stability | | 1101 | | (22) | |
| 62 | FASLG | | 356 | | Fas ligand (TNF superfamily, member 6) | | | 1q23 | | Induction of apoptosis | | 11936 | | (22) | |
| 63 | EGF | | 1950 | | Epidermal growth factor | | | 4q25 | | Growth, proliferation and differentiation of numerous cell types. | | 3229 | | (22) | |
| 64 | CDKN2A | | 1029 | | Cyclin-dependent kinase inhibitor 2A | | | 9p21 | | Tumor suppressor activity | | 1787 | | (22) | |
| 65 | PPARG | | 5468 | | Peroxisome proliferator- activated receptor gamma | | | 3p25 | | Pathology of numerous diseases including obesity, diabetes, atherosclerosis and cancer | | 9236 | | (22) | |
| 66 | PRKCI | | 5584 | | Protein kinase C, iota | | | 3q26.3 | | Necessary for BCL-ABL- mediated resistance to drug- induced apoptosis and therefore protects leukemia cells against  drug-induced apoptosis. | | 9404 | | (22) | |
| 67 | CCNE1 | | 898 | | Cyclin E1 | | | 19q12 | | Regulators of CDK kinases. | | 1589 | | (22) | |
| 68 | TNF | | 7124 | | Tumor necrosis factor | | | 6p21.3 | | Regulation of a wide spectrum of biological processes including cell proliferation, differentiation, apoptosis, lipid metabolism, and coagulation | | 11892 | | (22) | |
| 69 | CTSD | | 1509 | | Cathepsin D | | | 11p15.5 | | Pathogenesis of several other diseases, including breast cancer  and possibly Alzheimer’s dis- ease. | | 2529 | | (22) | |
| 70 | MMP2 | | 4313 | | Matrix metallopeptidase 2 | | | 16q12.2 | | Signal transduction | | 7166 | | (22) | |
